# Experimental validation and clinical feasibility of 3D reconstruction of coronary artery bifurcation stents using intravascular ultrasound

**DOI:** 10.1371/journal.pone.0300098

**Published:** 2024-04-16

**Authors:** Wei Wu, Akshat Banga, Usama M. Oguz, Shijia Zhao, Anjani Kumar Thota, Vinay Kumar Gadamidi, Vineeth S. Dasari, Saurabhi Samant, Yusuke Watanabe, Yoshinobu Murasato, Yiannis S. Chatzizisis

**Affiliations:** 1 Cardiovascular Division, Center for Digital Cardiovascular Innovations, University of Miami Miller School of Medicine, Miami, Florida, United States of America; 2 Department of Cardiology, Teikyo University School of Medicine, Tokyo, Japan; 3 Department of Cardiology and Clinical Research Center, National Hospital Organization Kyushu Medical Center, Fukuoka, Japan; Baylor Scott and White, Texas A&M College of Medicine, UNITED STATES

## Abstract

The structural morphology of coronary stents and the local hemodynamic environment following stent deployment in coronary arteries are crucial determinants of procedural success and subsequent clinical outcomes. High-resolution intracoronary imaging has the potential to facilitate geometrically accurate three-dimensional (3D) reconstruction of coronary stents. This work presents an innovative algorithm for the 3D reconstruction of coronary artery stents, leveraging intravascular ultrasound (IVUS) and angiography. The accuracy and reproducibility of our method were tested in stented patient-specific silicone models, with micro-computed tomography serving as a reference standard. We also evaluated the clinical feasibility and ability to perform computational fluid dynamics (CFD) studies in a clinically stented coronary bifurcation. Our experimental and clinical studies demonstrated that our proposed algorithm could reproduce the complex 3D stent configuration with a high degree of precision and reproducibility. Moreover, the algorithm was proved clinically feasible in cases with stents deployed in a diseased coronary artery bifurcation, enabling CFD studies to assess the hemodynamic environment. In combination with patient-specific CFD studies, our method can be applied to stenting optimization, training in stenting techniques, and advancements in stent research and development.

## 1. Introduction

Coronary artery stenting has transformed the field of interventional cardiology. The structural morphology of stents, including aspects such as stent expansion, tissue scaffolding, strut malapposition, side branch jailing, and strut fracture, along with the local hemodynamic environment after stent deployment in coronary arteries, play pivotal roles in determining the procedural success and subsequent clinical outcomes [1–7]. High-resolution intracoronary imaging employing high-definition intravascular ultrasound (IVUS) and optical coherence tomography (OCT) has become a crucial tool in stenting optimization [8]. However, while cross-sectional imaging of deployed stents by IVUS or OCT provides valuable data on stent morphology, it fails to provide a holistic view of the stent’s spatial distribution relative to the lumen and the local hemodynamic environment within the stent (macroenvironment) and around the stent struts (microenvironment). To address these considerations, an accurate three-dimensional (3D) reconstruction of coronary stents, paired with the 3D reconstruction of the arterial lumens, can offer valuable information to optimize stenting procedures in cardiac catheterization laboratories, conduct patient-specific computational fluid dynamics (CFD) simulations post-stenting, and support the advancement of new stent technologies through research and development. There have been very few attempts at IVUS-based 3D reconstruction of coronary stents [9–13], but none have successfully built a complete 3D stent reconstruction model. Furthermore, there have been no CFD simulations on the IVUS-based stent-implanted lumen model.

This study aims to achieve the following objectives: i) To introduce a novel IVUS-based algorithm for 3D reconstruction of coronary stents with bifurcations, ii) To experimentally examine the accuracy (at strut level) and reproducibility of the algorithm using patient-specific silicone coronary bifurcation models, iii) To evaluate the feasibility of implementing the algorithm for a clinical case where a stent was implanted in a diseased coronary bifurcation and iv) To investigate the potential of conducting CFD studies on the 3D reconstructed stent and bifurcation model of the clinical case.

## 2. Methods

### 2.1 Study participant details

We retrospectively selected n  =  1 left main bifurcation IVUS and angiography data from a database of Kyushu Medical Center, Fukuoka, Japan. The use of the IVUS data was reviewed and approved by the ethics committee of the National Hospital Organization Kyushu Medical Center (Institutional Review Board #20C035). All methods were carried out in accordance with relevant guidelines and regulations, and written informed consent was obtained from the subjects. The database included only adult patients. The anonymized data was accessed on April 10th, 2023.

### 2.2 Experimental studies

#### 2.2.1 Experimental coronary bifurcation models, flow chamber studies and imaging procedures

**Fig 1:**
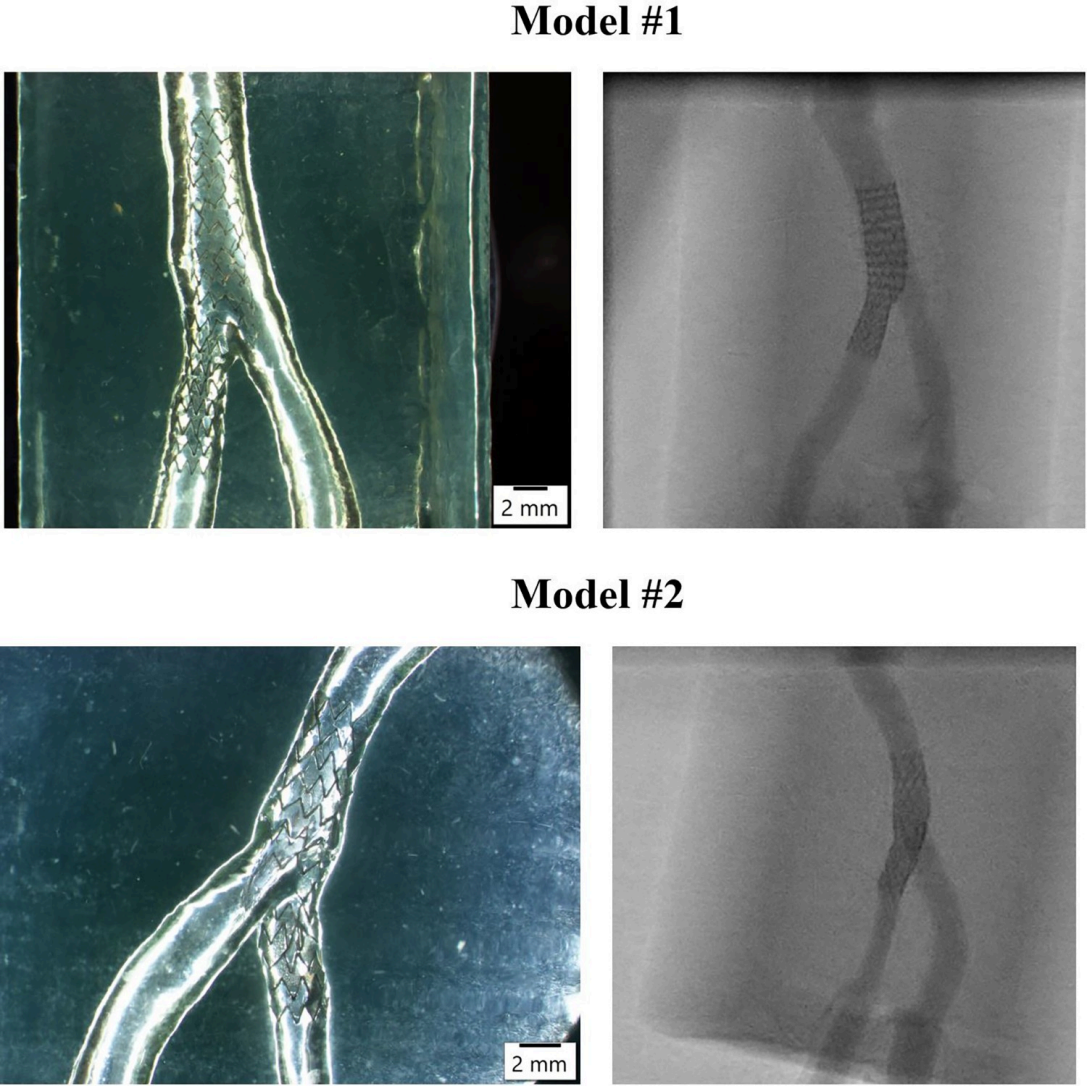
Patient-specific silicone bifurcation Model #1 and #2 for IVUS reconstruction method validation. After the stents were implanted in the silicone models, the stereomicroscope (left) and angiography (right) imaging were obtained.

Two patient-specific silicone models of coronary artery bifurcation were created (**Fig 1**) using our in-house technique as previously described [14]. In brief, the 3D lumen geometries were created in 3D CAAS Workstation 8.2 (Pie medical imaging, Maastricht, The Netherlands) using two angiographic projections. Negative molds were designed according to the geometries and were 3D printed with acrylonitrile butadiene styrene. Polydimethylsiloxane was mixed with its curing agent and then poured into molds. After curing, the silicone models were moved to an acetone beaker to dissolve the acrylonitrile butadiene styrene material. The finished silicone models were placed in a custom-made flow chamber. A bioreactor circuit was connected to the inlet and outlets of the flow chamber, allowing a closed circuit with a continuous flow of 750 ml blood-mimicking fluid (Doppler test fluid, CIRS, Norfolk, Virginia, USA) at a constant 100 ml/min flow rate at room temperature. Two different widely used second-generation stents (Megatron and Synergy stent, Boston Scientific, Maple Grove, MN, USA) with a nominal diameter of 4.0 mm were directly implanted into the silicone Model #1 and #2, respectively (**Table 1**). For Model # 1, the Megatron stent was implanted across a big left main (LM) (about 5mm diameter) and the left anterior descending artery (LAD) to assess the ability of our methodology to reproduce strut malapposition.

**Table 1:** Experimental stent types and implanting parameters.

	MV	SB	Stent and implantation	Post-dilation
Model #1	LAD	D1	Megatron 4.0*20 mm @ 12 atm	Stent balloon @ 20 atm
Model #2	LAD	D2	Synergy 4.0*16 mm @ 11 atm	NA

LAD: Left anterior descending artery; D1: Diagonal 1; D2: Diagonal 2; NA: Not applicable.

**Fig 2:**
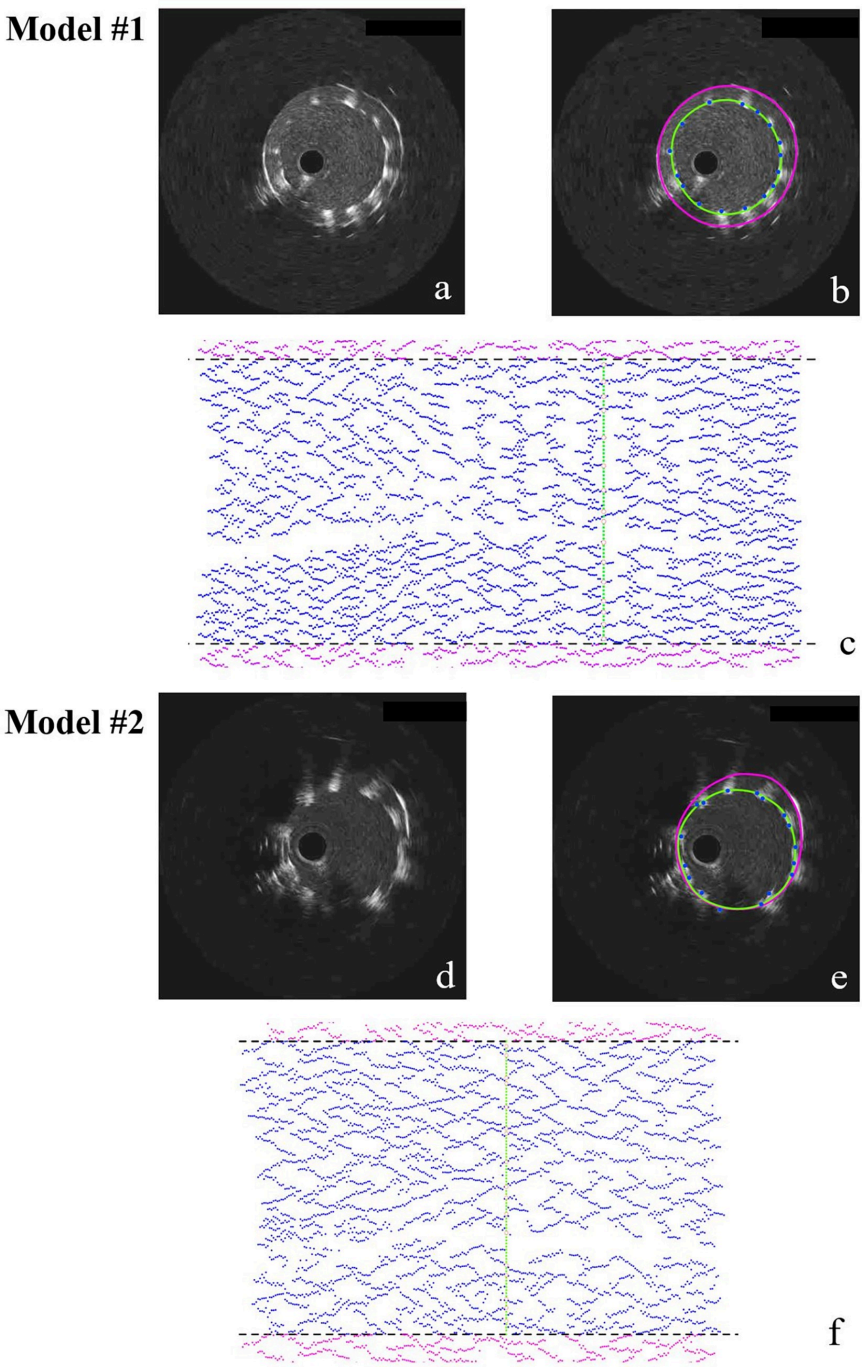
IVUS imaging segmentation for Model #1 and #2, and 2D unrolling of segmentation. Based on the original IVUS images **(a and d)**, the segmentations **(b and e)** include lumen (purple curves), stent contour (green curves), and strut points (blue points). In 2D unrolling of segmentation **(c and f)**, the purple points are the extension of the stent struts (blue points) to show the structural repeatability, and the black hiding lines represent the unrolling boundary. The segmented stent contour and struts **(b and e)** are highlighted in the 2D unrolling.

The stented silicone models were imaged with angiography at two projections with at least a 30° difference in viewing angles to obtain the centerlines of the main vessel (MV) and side branch (SB) using CAAS as mentioned above and VMTK (Orobix, Bergamo, Italy). IVUS imaging of the stented models was obtained with Opticross 6 HD, 60 MHz catheter (Boston Scientific, Marlborough, MA, USA). The imaging catheter with an axial resolution of approximately 22 μm [15] was advanced using a 6F guiding catheter, and an automatic pullback was performed at a constant speed of 1.0 mm/sec, yielding a frame distance of 0.033 mm. All the pullback frames were analyzed offline, and the lumen sections were semi-automatically segmented using EchoPlaque 4.0 (INDEC Medical Systems, Los Altos, CA, US) (**Fig 2A, 2B, 2D and 2E**).

#### 2.2.2 3D reconstruction of silicone lumen

The technique for semi-automatic 3D reconstruction of the silicone bifurcation was described in detail in our relative work and summarized in **Table 2** [16]. Briefly, the centerlines obtained in the previous step served as the backbone of the bifurcation reconstruction. The lumen contours from the segmented IVUS images were aligned along the centerline and oriented using the bifurcation carina as a reference. The aligned lumen contours were lofted to create the MV and SB surfaces, which were merged and smoothed to build the final 3D bifurcation surface.

**Table 2:** Steps of the proposed method for IVUS 3D stent reconstruction.

1. 3D lumen reconstruction	2.1. Stent contour and strut point segmentation in IVUS images
	2.2. Stacking, orientation, straightening, and planar unrolling of strut points and stent contours
	2.3. 2D stent pattern matching
3.1. Rolling back of the planar stent pattern with the 3D reconstructed lumen as reference	
3.2. Stent volume extrusion	

IVUS: Intravascular ultrasound; 2D: 2-dimensional; 3D: 3-dimensional.

#### 2.2.3 IVUS strut segmentation and two-dimensional strut unrolling

The steps for strut segmentation and two-dimensional (2D) strut unrolling are illustrated in **Fig 2**. We manually identified the stent contours and strut points using EchoPlaque. A significant challenge in IVUS strut segmentation is the artifacts in the IVUS signals. These artifacts often lead to larger strut signal sizes than the actual struts and overlap close signals, complicating accurate strut localization for operators. To address this, we have adopted a uniform segmentation approach, as depicted in **Fig 2A, 2B, 2D and 2E**. We identified the segmented strut points at the middle of the luminal edge of the signals, ensuring a more precise and consistent determination of strut positioning. The segmented stent contours and strut points were imported into Grasshopper 3D, a plug-in within Rhinoceros 3D (Robert McNeel & Associates, Seattle, WA, USA). Then, they were stacked in a straight line along the IVUS catheter center, oriented with the lumen contour at the same IVUS frame, and finally unrolled on a 2D plane (**Fig 2C and 2F**). For the convenience of viewing, only one stent contour was shown in the 2D unrolling.

#### 2.2.4 Matching 2D stent pattern to unrolled strut points

**Fig 3:**
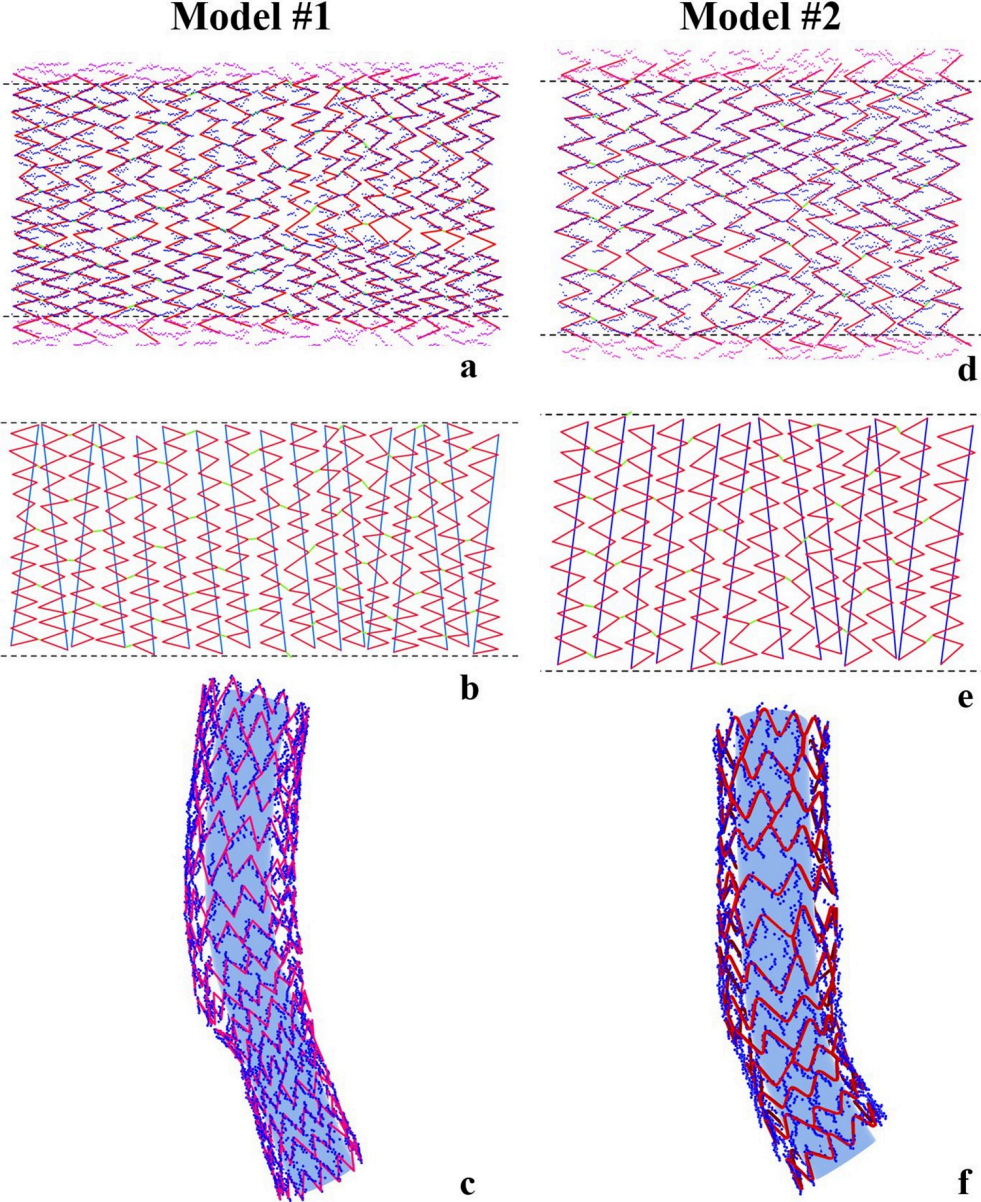
2D stent pattern matching and rolling back for silicone models. The matched 2D stent patterns **(a and d)** are trimmed at unrolling boundaries and then connected at end vertices **(b and e)** to ensure accuracy when rolled back. The stent pattern is rolled back into 3D space and overlapped with strut segmentation in 3D **(c and f)**. The blue tubes inside stent models do not depict reconstructed lumen but facilitate better visualization of stent struts.

For each model, a 2D stent pattern was matched to the unrolled strut points with the reference of the planar stent design, which was kindly provided by the stent manufacturer. From the previous segmentation step, we could see there were still many messy strut points (**Fig 2C and 2F**). To overcome this difficulty, the following rules were applied to the stent pattern matching (**Fig 3A and 3D**): **i**) The stent pattern is composed of “zigzag” polylines (red) connected by single-line links (green). Each polyline represents a circularly closed stent strut in 3D and should cover as many 2D strut points in its range while following the stent design. The vertex of the line segments of the polyline should be at the “peak” or “valley” of the strut points. **ii**) The location and the sequence of the links are consistent with the planar stent design. **iii**) The operator bridges the gaps of strut points due to guidewire shadow, following the stent pattern. **iv**) At the unrolling boundaries, the line segments of each polyline are repeatable to maintain the stent structure continuity.

#### 2.2.5 Trimming and connection of stent pattern

To further ensure the structural continuity of the rolled-back stent pattern, any line segments crossing the unrolling boundaries were trimmed, and the two end vertices of each trimmed polyline were connected by a single line (**Fig 3B and 3E**).

#### 2.2.6 3D stent reconstruction

The completed 2D stent pattern (polylines, links, and connecting lines) was rolled back to 3D space with the reference of previously reconstructed bifurcation lumen. First, the two vertices of each line segment were rolled back to 3D space in pairs. Then, the rolled-back vertices are connected in pairs to re-create the polylines, links, and connecting lines in 3D space. Each connecting line will restore its correct length in 3D and accurately build a circularly closed strut pattern with the polyline (**Fig 3C and 3F**). After the round of the stent pattern at the vertices, the final strut volume was created by extruding the rectangular stent sections along the 3D reconstructed stent pattern.

#### 2.2.7 Micro-computed tomography (μCT) and stereoscopic imaging

The μCT imaging of the stented silicone models was used as a reference for the validation of the stent reconstruction algorithm. μCT imaging (Skyscan 1172 version 1.5, Antwerp, Belgium) was performed with the following parameters: Image pixel size 26.94 μm, voltage 100 kV, current 100 μA, and slice thickness 27 μm. The stented models were 3D reconstructed from μCT images (Mimics 22.0, Materialise, Leuven, Belgium) and then smoothened (Meshmixer, Autodesk Research, New York, NY, USA). Stereoscopic imaging was performed with an Olympus SZX16 camera (Tokyo, Japan) using a 6X magnification factor.

#### 2.2.8 Experimental validation of the 3D stent reconstruction algorithm

The IVUS-based 3D reconstructed stents were compared morphologically to the μCT reconstructed ones. Three metrics were used for the quantitative comparisons between IVUS-based and μCT-based reconstructions: **i)** Mean stent diameter (MSD), defined as the average stent diameter of serial cross-sections every 0.1 mm along the stent axial direction, **ii)** Stent length and **iii**) stent malapposition. As for MSD, we observed a consistent difference in lumen diameter between IVUS and μCT (**S1 Fig**) because IVUS measurement was found to overestimate the actual lumen size [17]. Thus, the lumen diameters were normalized using the z-score to account for the systemic discrepancy in lumen size between the IVUS and μCT [18]. The z-score is a statistical measurement that describes a value’s relationship to the mean of a group of values, measured in terms of standard deviations from the mean. MSD’s z-score was calculated by subtracting the mean from the values of MSD and then dividing the result by the standard deviation. The formula for calculating a z-score is:

$$Z=\frac{\left(X-\mu \right)}{\sigma }$$ (1)

where:

X is the value of MSD; μ is the mean of the MSD; σ is the standard deviation of the MSD.

As for stent malapposition, we took five representative sections with malapposition at the same locations for IVUS and μCT reconstruction. The malapposition areas (the area between stent struts and lumen boundary) were compared morphologically, and the malapposition area ratios (ratio between malapposition area and lumen area) were calculated.

To minimize possible biases, different operators performed the 3D reconstruction from IVUS, 3D reconstruction from μCT, and comparison between IVUS- and μCT-based models.

#### 2.2.9 Reproducibility

To assess the reproducibility of the IVUS-based 3D reconstruction method, the stents were 3D reconstructed by two independent operators. The reconstructed stents were compared in terms of morphology and MSD as mentioned above.

### 2.3 Clinical studies

#### 2.3.1 Clinical feasibility, processing times, and CFD studies

**Fig 4:**
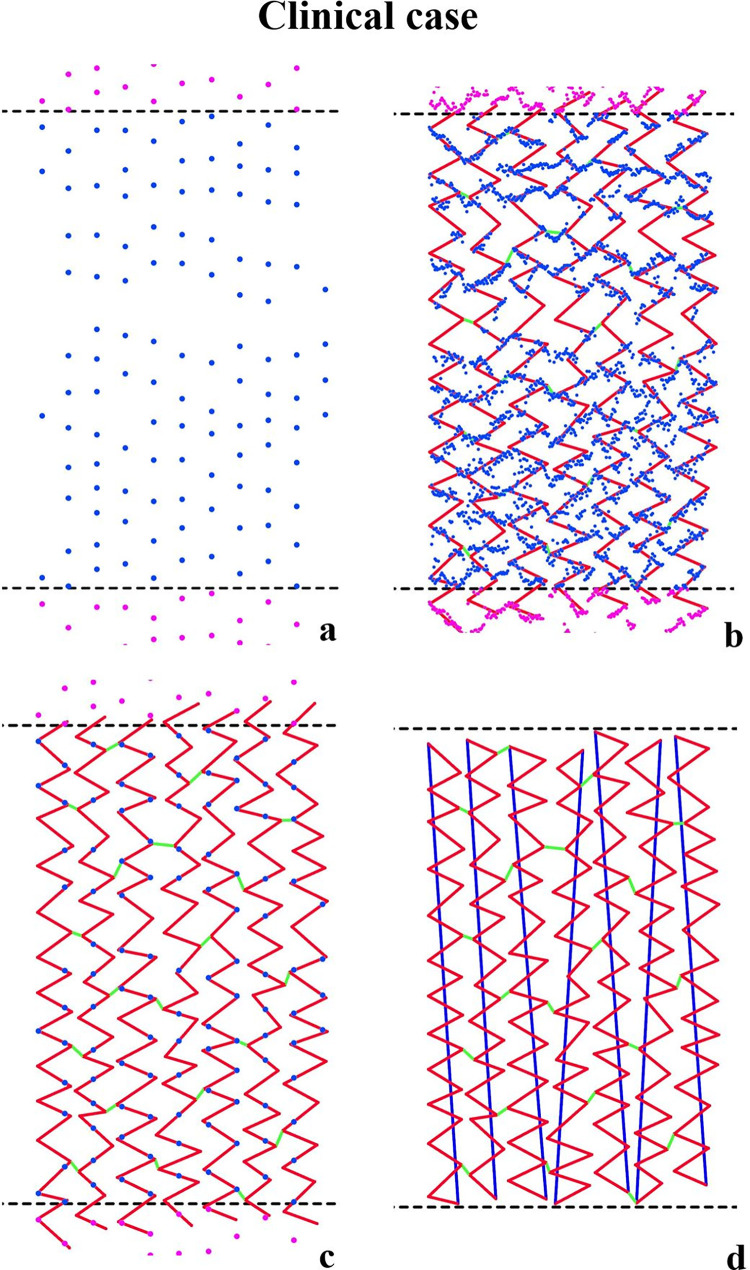
2D stent pattern matching for the clinical case. The 2D segmented stent points after ECG gating **(a).** 2D stent pattern was matched with the guidance of all segmented stent points **(b).** The matched stent pattern is stuck to the ECG-gated stent points **(c)**. The stent pattern was trimmed and connected **(d)**.

The clinical feasibility of our stent reconstruction method and processing times were assessed in a patient’s coronary artery bifurcation. This patient had one stent (Megatron 4.0*8 mm) implanted in the proximal left main vessel with the aorto-ostial lesion. Both IVUS and angiography data were acquired according to the imaging protocols mentioned in section 2.1. To minimize the twisting and swinging (lateral and longitudinal motions of the catheter) effects in the clinical IVUS scanning, we applied electrocardiogram (ECG)-gating to obtain the IVUS frames at the end-diastolic phase for lumen reconstruction [16]. However, after ECG-gating, the segmented stent points lost most information and were not enough for 2D stent pattern matching (**Fig 4A**). Thus, we used all 2D stent points to guide the pattern matching (**Fig 4B**) and kept the matched pattern sticking on the ECG-gated stent points (**Fig 4C**). After trimming and connecting the matched stent pattern (**Fig 4D**), the lumen and stent were 3D reconstructed using the steps outlined in our proposed algorithm. We evaluated the reconstruction morphology using angiography and a ’mapping-back’ technique [16]. This technique involves creating a section of the reconstructed stent and lumen and mapping this section back to the IVUS frame at the exact location. Furthermore, another independent operator repeated the 3D stent reconstruction to assess the method’s reproducibility. The two stent models were compared in terms of morphology and MSD. Finally, we calculated the processing time required for each step to gauge the time efficiency of our stent reconstruction method.

We evaluated the feasibility of conducting CFD studies using lumen and stents reconstructed with our method. The fluid domain was discretized into polyhedral elements, with an element size of 0.15 mm for the lumen and 0.03 mm for the stent. We employed velocity inlet and outflow ratios as boundary conditions, using pulsatile flow data from a human left coronary artery [19], and the inlet velocity was tuned according to inlet diameter [20]. The outflow ratio was determined based on the diameter ratio of MV and SB [20]. To minimize the effects of boundary conditions, we added extensions with lengths ten times the diameters of the inlet and outlet sections. We treated blood as a laminar Newtonian fluid, with a density of 1,050 kg/m3 and dynamic viscosity of 0.0035 Pas. Simulations were conducted for n = 3 cardiac cycles with a time step of 0.01 s, and only the results from the last cycle were displayed. We calculated the time-averaged wall shear stress (TAWSS), relative residence time (RRT), and wall shear stress gradient (WSSG) using the following equations:

$$TAWSS=\frac{1}{T}\int_{0}^{T}|\tau_{w}|dt$$ (2)

$$RRT=\frac{1}{TAWSS(1-2OSI)}$$ (3)

$$WSSG=\alpha_{x}\frac{\partial \tau_{w}}{\partial x}+\alpha_{y}\frac{\partial \tau_{w}}{\partial y}+\alpha_{z}\frac{\partial \tau_{w}}{\partial z}$$ (4)

Where:

$$OSI=0.5(1-\frac{\left|\int_{0}^{T}\tau_{w}dt\right|}{TAWSS})$$ (5)

$$\alpha =\frac{\tau_{w}}{\left|\tau_{w}\right|}$$ (6)

and *τ_w_* is the wall shear stress, and *α* is the wall shear stress direction.

### 2.4 Quantification and statistical analyses

For experimental validation and reproducibility studies, bland-Altman analyses were performed with the statistical package GraphPad Prism 8.0 (GraphPad Inc., San Diego, CA, USA).

## 3. Results

### 3.1 Experimental validation

#### 3.1.1 Stent and lumen morphology

**Fig 5:**
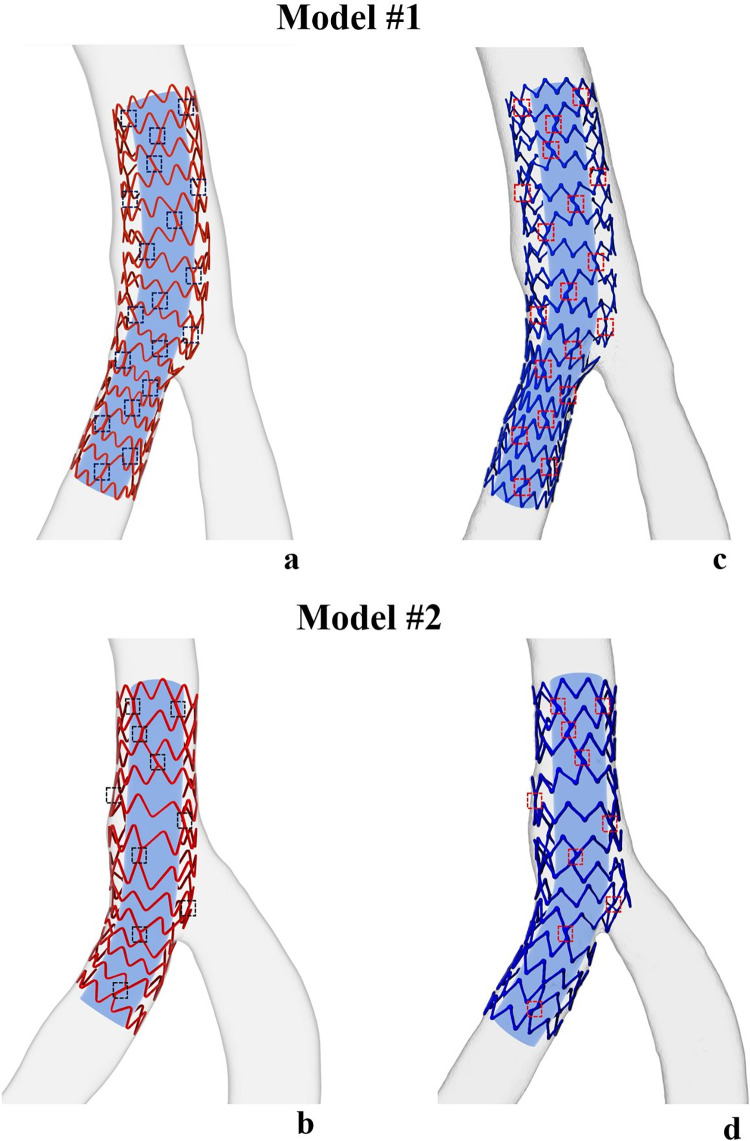
Morphological comparison between 3D IVUS stent reconstruction (a and b) and μCT reconstruction (c and d). The strut links are indicated by boxes to show the agreement of reconstruction. The blue tubes inside stent models do not depict reconstructed lumen but facilitate better visualization of stent struts.

The experimental validation successfully demonstrated 3D IVUS reconstruction of the stents implanted in the silicone models. The morphology of these reconstructed stents and bifurcations was compared with their respective 3D models reconstructed using μCT (**Fig 5**). Each model displayed remarkable consistency in morphology between the stents and bifurcations reconstructed using IVUS and those produced via μCT. Specifically, the dotted squares in **Fig 5** indicate substantial agreement regarding the link count, 3D positioning, and orientation of stent links between the stents reconstructed using IVUS and μCT. For 3D IVUS reconstruction, Models #1 and #2 had 48 and 30 cross-links, respectively, which exactly agreed with the cross-links from 3D μCT reconstruction. Furthermore, the thickness of the 3D IVUS reconstruction precisely represented the thickness of the real stent, given that the volume was extruded utilizing an actual strut cross-section. In contrast, the μCT reconstruction thicknesses fluctuated around the true strut dimension because the volume was reconstructed directly from μCT data. This data suggests that the original stent structure, including detailed features at the level of individual struts was reliably recreated by our 3D IVUS reconstruction methodology.

#### 3.1.2 Stent diameter, length, and malapposition

**Fig 6:**
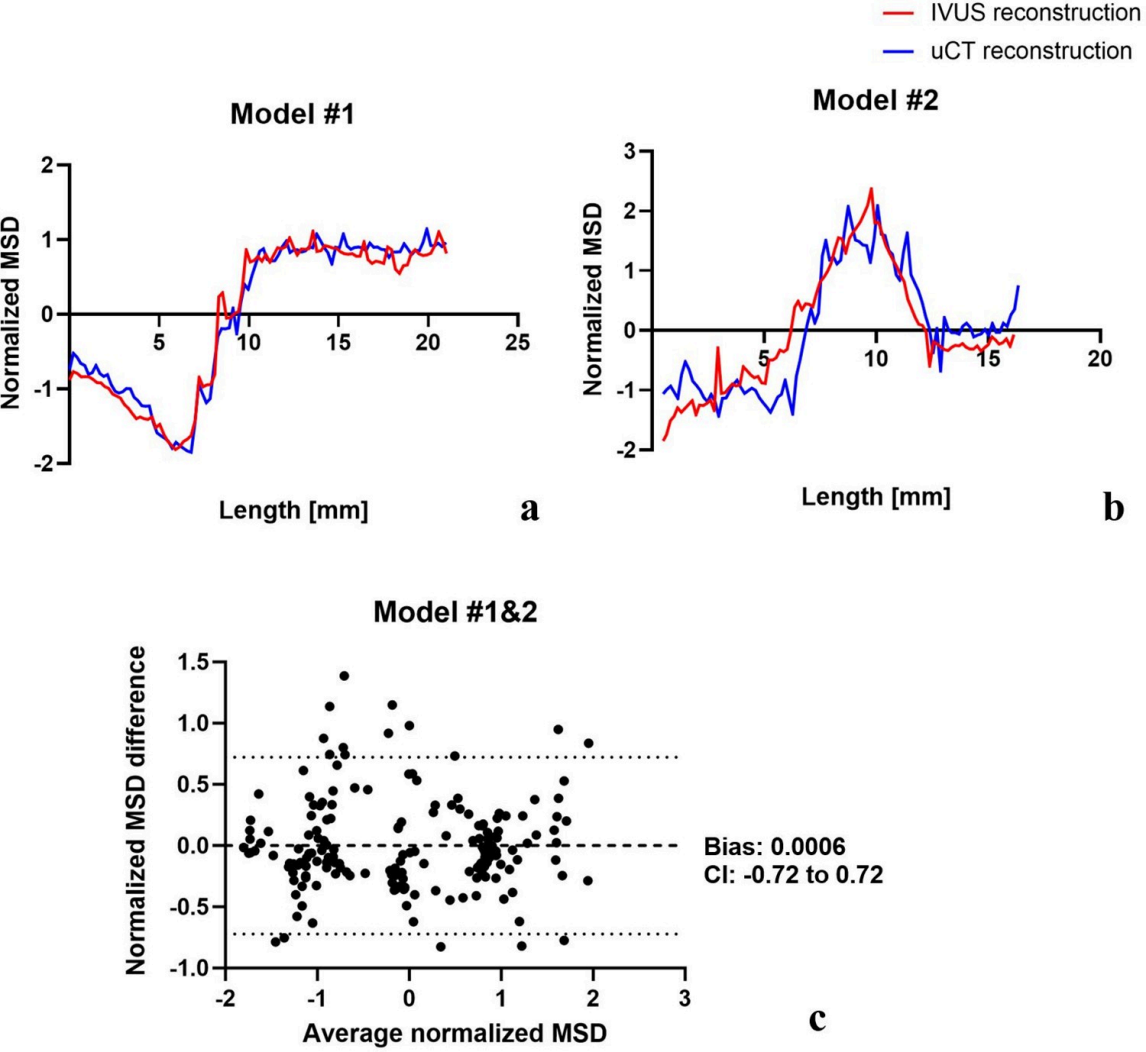
Normalized mean stent diameter (MSD) comparison between IVUS 3D reconstruction and μCT reconstruction.

**Fig 7:**
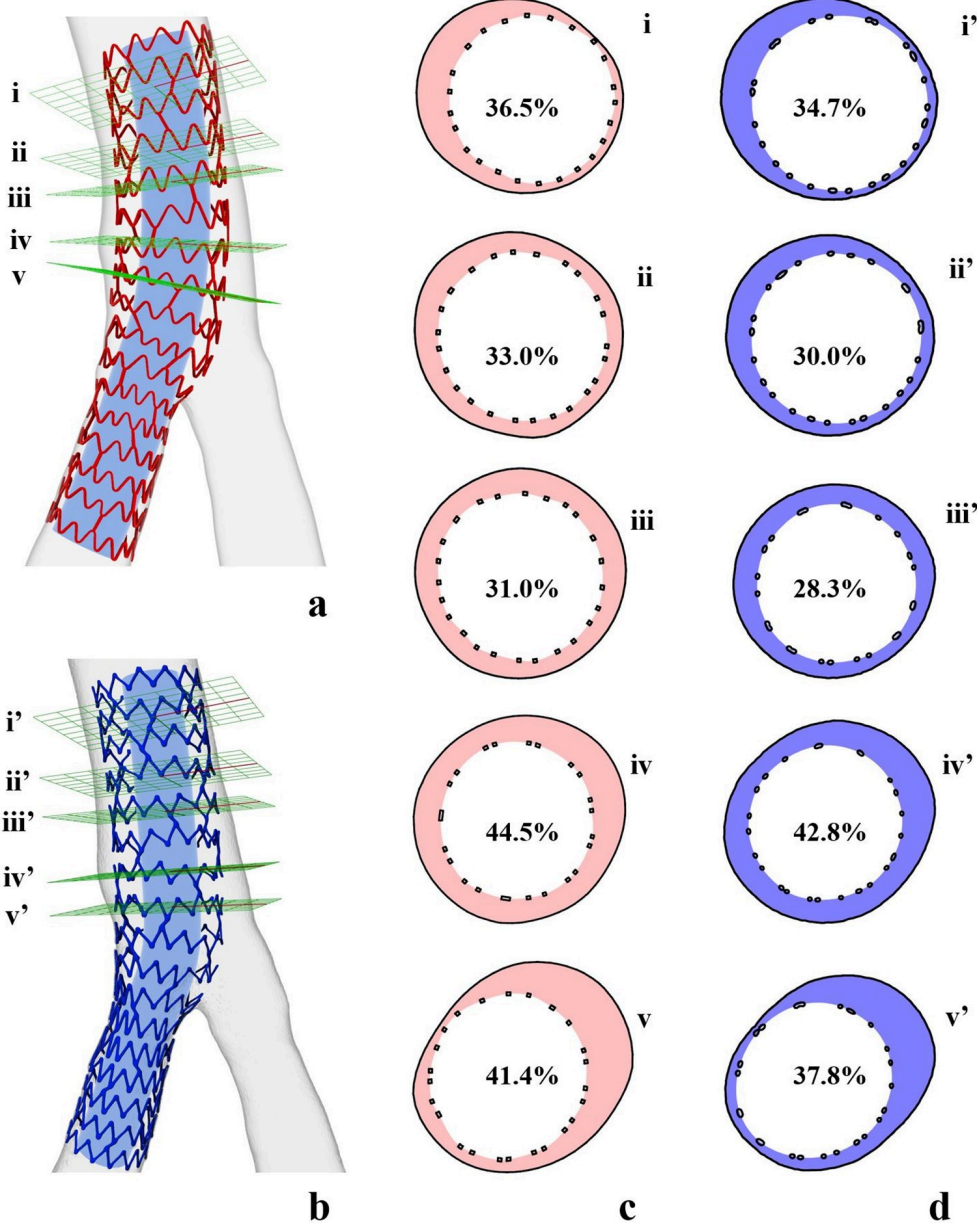
Stent malapposition comparison of Model #1 between IUVS and μCT reconstruction. IVUS reconstructed model **(a)** and μCT reconstructed model **(b)** have respective section planes i-v and i’-v’ at the same locations. These sections of IVUS **(c)** and μCT reconstruction **(d)** have colored malapposition areas, and the percentage numbers indicate the malapposition area ratios. The blue tubes inside stent models do not depict reconstructed lumen but facilitate better visualization of stent struts.

The normalized mean stent diameter (MSD) displayed strong agreement between the stent models reconstructed by IVUS and those reconstructed by μCT (**Fig 6A and 6B**). The Bland-Altman analysis, conducted to assess the consistency of the normalized MSD between the stents reconstructed through IVUS and μCT, demonstrated an insignificant mean difference of 0.0006 (CI -0.72 to 0.72) (**Fig 6C**). As for the stent length, Model #1 was measured as 21.9 mm (IVUS) and 21.2 mm (μCT), and Model #2 was measured as 16.3 mm (IVUS) and 16.5 mm (μCT). This data suggests strong alignment in stent length between the 3D stent reconstructions derived from IVUS and μCT, reinforcing the high accuracy of our method. The evaluations of stent malapposition are depicted in **Fig 7**. For each pair of section planes at the same location, the malapposition areas from IVUS and μCT had close agreement in morphology and ratio, indicating an accurate assessment of stent malapposition.

#### 3.1.3 Reproducibility

**Fig 8:**
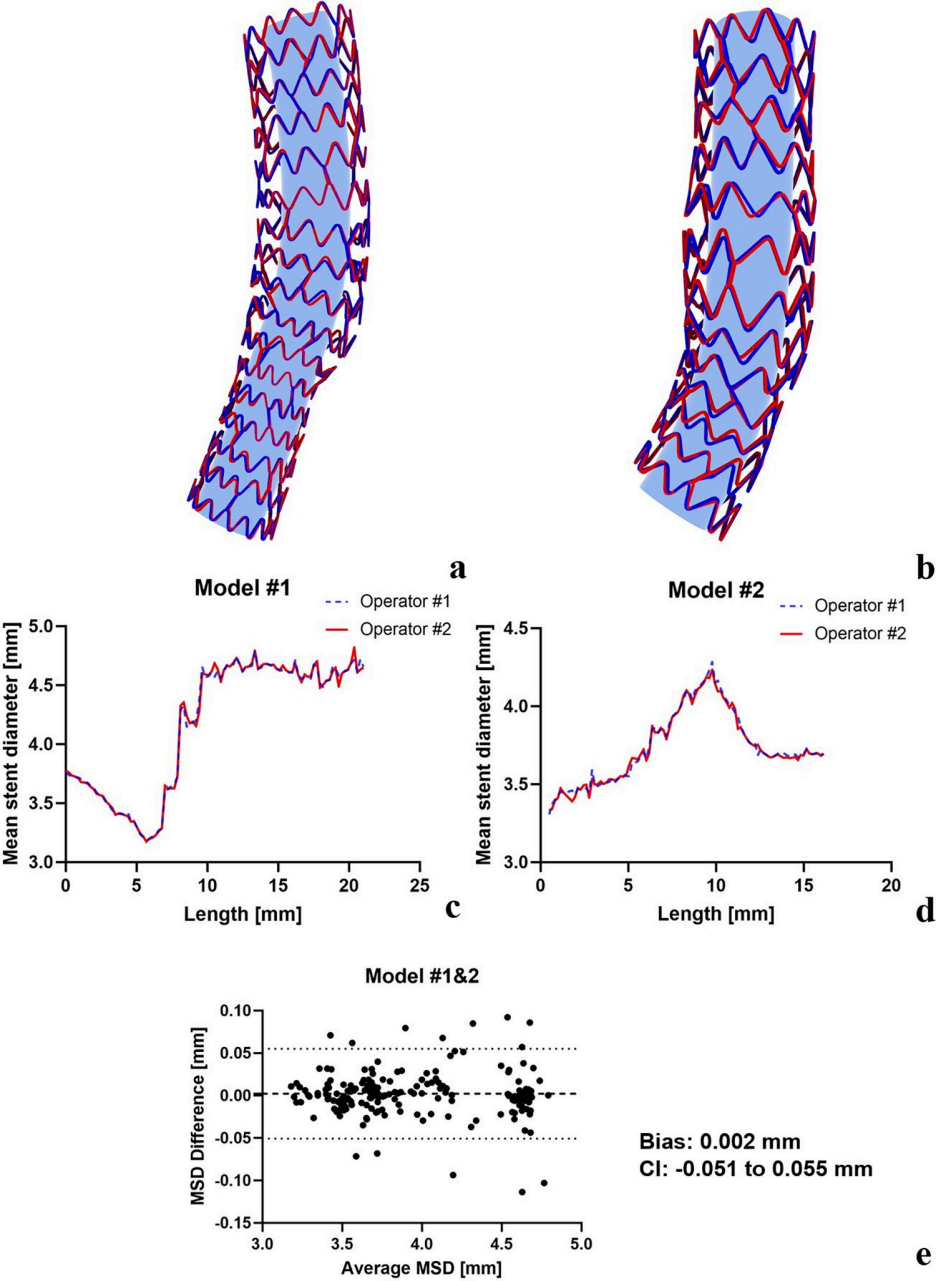
Reproducibility analysis, including morphological (a and b) and diameter measurement comparison (c, d, and e). The blue tubes inside stent models do not depict reconstructed lumen but facilitate better visualization of stent struts.

Our method’s reproducibility was distinctly demonstrated through the overlapping of the 3D reconstructed stents completed by two independent operators, as depicted in **Fig 8A and 8B**. This overlay confirms the substantial level of inter-observer reproducibility inherent in our technique. Upon quantitative comparison of the MSD of the reconstructed stent models created by these two independent operators, the resulting curves, which track the MSD along the stent’s axial direction, were nearly identical (**Fig 8C and 8D**). Additional evidence of our method’s high reproducibility was provided by a Bland-Altman analysis conducted on both models (**Fig 8E**), revealing an insignificant mean difference in MSD of 0.002 mm (CI -0.051 mm to 0.055 mm). These results robustly affirm the excellent reproducibility of our proposed stent reconstruction technique.

### 3.2 Clinical feasibility and CFD studies

**Fig 9:**
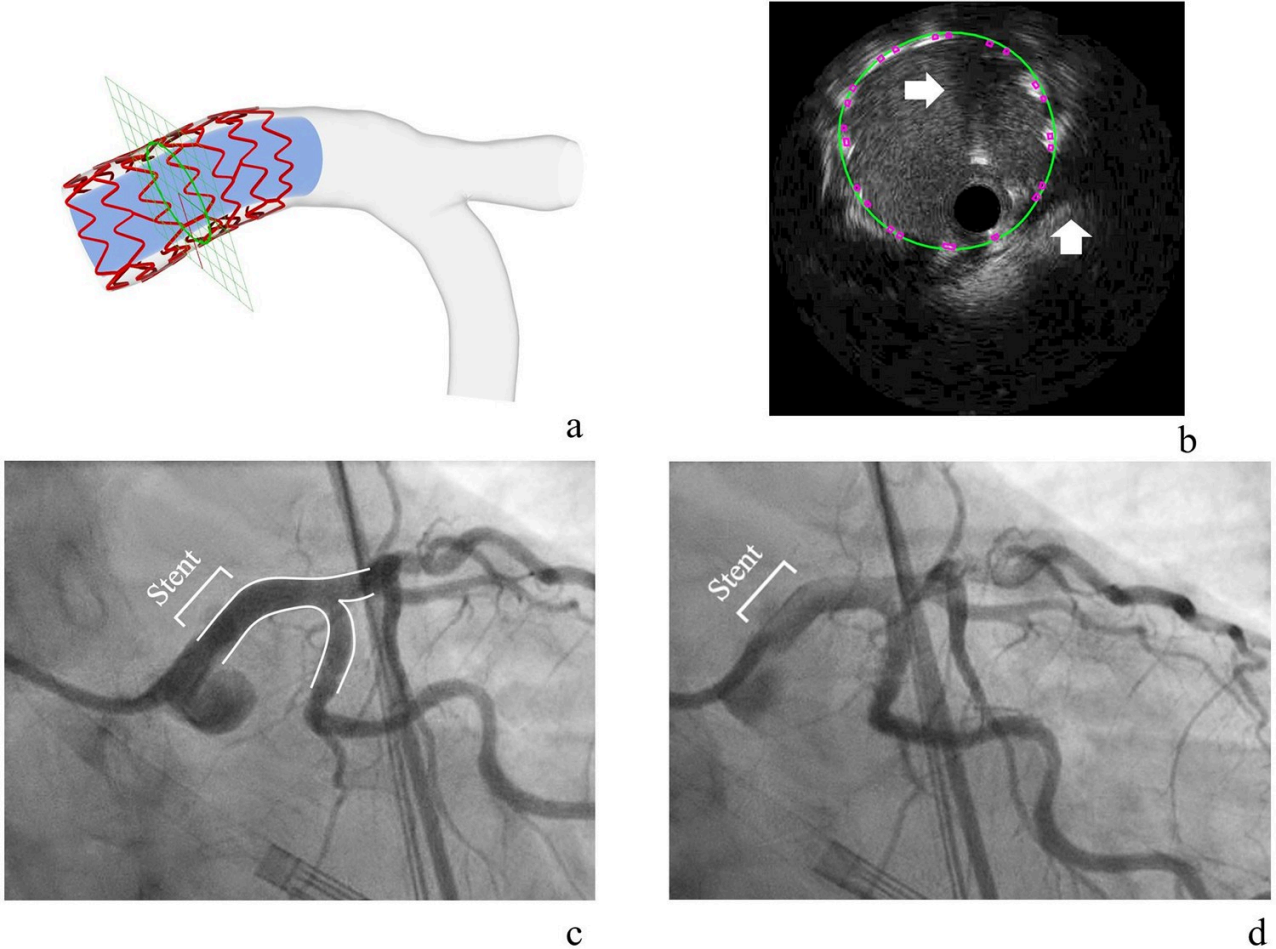
3D IVUS stent reconstruction of the clinical case. After trimming and connection, the 2D stent pattern was rolled back to reconstruct the 3D stent model with the reconstructed bifurcation lumen **(a)**. The final 3D reconstructed clinical case checked with the mapping-back technique **(b)** and angiography **(c and d)**. In the mapping-back section, the green curve and purple squares are sections of the reconstructed lumen and stent struts, respectively. The two white arrows indicate the guidewire shadows. The blue tube inside stent model does not depict reconstructed lumen but facilitates better visualization of stent struts.

In the clinical case of stent implantation in the LM bifurcation, the 2D stent pattern was matched to the unrolled segmented strut points, referencing the corresponding planar stent design. This pattern was then trimmed and linked, leading to a successful 3D reconstruction of the stent model and bifurcation (**Fig 9A**). The model section check, conducted via mapping-back, confirmed that the reconstruction accurately reproduced the size and location of the actual lumen and stent struts, respectively (**Fig 9B**). Interestingly, the reconstruction appeared to ’compensate’ for the stent struts obscured by two guidewire shadows during the IVUS scanning process. The reconstructed stented bifurcations were qualitatively compared with the angiograms, revealing a good agreement in shape (**Fig 9C and 9D**). The reconstructed models from two independent operators had high reproducibility, as shown in **S2 Fig**. The two models had compatible overlay, nearly identical MSD curves, and the Bland-Altman analysis showed an insignificant mean difference of -0.001 mm (CI -0.021 mm to 0.020 mm) in MSD. The processing times for each stage, ranging from image processing to the final 3D lumen and stent reconstruction, are detailed in **Table 3**. The time taken for the 3D reconstruction of stents was approximately 200 minutes.

**Table 3:** Processing times for 3D stent reconstruction of the clinical case.

**Steps**	**Time (min)**
**Step 1. 3D lumen reconstruction**	50
**Step 2. 2D stent reconstruction**	
1. IVUS segmentation of stent contours and strut points	120
2. Data importing and parameter setting	5
3. Segmentation stacking, orientation, straightening, and planar unrolling	5
4.Planar stent pattern matching	40
**Step 3. 3D rolling back and stent volume creation**	30
*Total time for 3D stent reconstruction (excluding step 1)*	**200**
*Total time for the whole process*	**250**

IVUS: Intravascular ultrasound; 2D: 2-dimensional; 3D: 3-dimensional.

**Fig 10:**
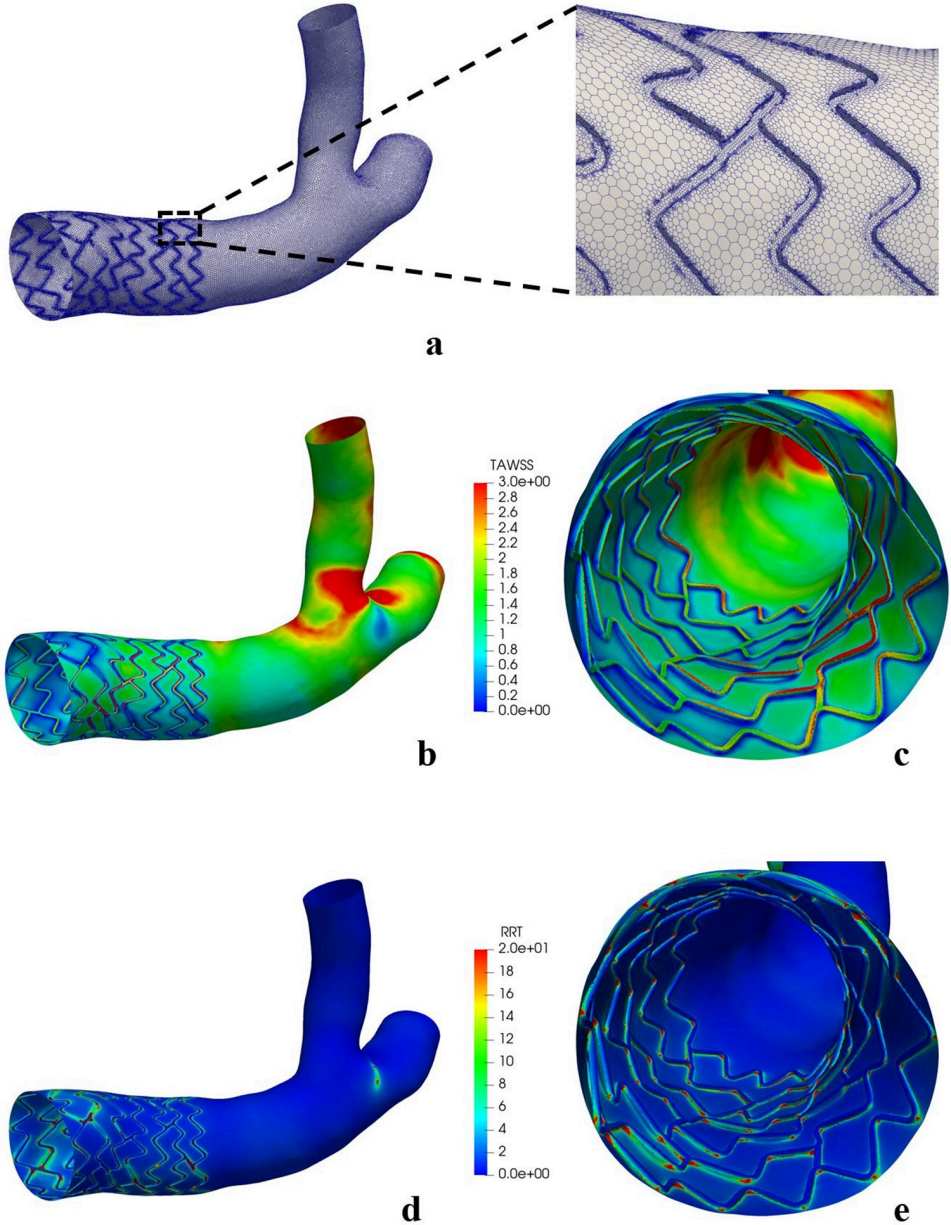
CFD simulation on the 3D clinical reconstruction. **(a)** The polyhedral meshing of the stented bifurcation model. Distribution of TAWSS (Pa) and RRT (Pa^-1^) in macroenvironment **(b and d)** and microenvironment **(c and e)**. TAWSS: time-averaged wall shear stress; RRT: Relative residence time (RRT).

Following the reconstruction, the stented bifurcation was subjected to CFD analyses using polyhedral meshing (**Fig 10A**). These analyses revealed the distribution of TAWSS and RRT across the reconstructed stented bifurcation, also known as the macroenvironment (**Fig 10B and 10D**). Upon closer examination at the strut level, or microenvironment, we observed that the areas near the stent-lumen interface, where turbulent flow occurred, exhibited decreased TAWSS values and increased RRT values (**Fig 10C and 10E**). The WSSG results are displayed in **S3 Fig**.

## 4. Discussion

This study presents a pioneering approach for the 3D reconstruction of coronary stents, leveraging a combination of IVUS and angiography imaging techniques. Our research primarily concentrates on two aspects: **i**) Evaluating the accuracy and reproducibility of our proposed method using patient-specific silicone models of coronary bifurcations, and **ii**) Assessing the clinical feasibility, processing time, and potential for conducting CFD studies on clinical bifurcation case. For validation purposes, we used μCT reconstructed models as the gold standard. Our findings demonstrate that our proposed algorithm accurately duplicates the complex 3D stent architecture at a micro-scale (strut level) with exceptional reproducibility. Moreover, our results suggest that it is feasible for clinical applications where stents are deployed in diseased coronary bifurcations. This feasibility allows us to conduct CFD studies, offering an analysis of the hemodynamic environment on both macro- and micro-environmental levels.

The undertaking of 3D stent reconstruction based on IVUS scanning has posed significant challenges for nearly two decades, beginning with the initial studies of Mentz et al [10] and Terashima et al [11]. However, their studies focused on simply rendering stent segmentations to exhibit an approximate structure of the implanted stents. These renderings cannot be used for further CFD analysis. Recently, only a few studies have aimed to automate the detection and segmentation of stent struts from IVUS images [9,12,13]. However, none have successfully created a complete stent model based on IVUS images, let alone the model with both the stent and implanted bifurcation. To the best of our knowledge, this is the first study to achieve a comprehensive 3D stent and coronary bifurcation reconstruction based on IVUS imaging. Moreover, we provide robust validation of our method for the in-vitro models and applied the methodology to a clinical case.

This proposed approach incorporates numerous technical aspects and innovations from our previous research on OCT-based stent reconstruction [21], and IVUS-based lumen reconstruction [16]. These aspects include accurate coronary artery lumen 3D reconstruction with good reproducibility, the 2D unrolling of strut points to bypass the challenges associated with direct 3D reconstruction, the identification of stent link locations as essential references for precise stent pattern matching, and the utilization of the visual programming environment (Grasshopper) to automate the reconstruction process and enhance both accuracy and reproducibility. Additionally, compared to the existing OCT-based 3D stent reconstruction works (based on our knowledge, there are no completed IVUS-based 3D stent reconstruction works, and the OCT-based stent 3D reconstructions have multiple similarities to our IVUS-based study), three other significant aspects of this study are noteworthy: First, integrating the 2D stent pattern at the unrolling boundaries during rolling back has always been a complex issue for stent reconstruction methods that utilize a 2D strategy [21]. In our method, the process of trimming and connecting the 2D stent pattern at the unrolling boundaries allowed for a seamless integration of the strut structure when the polylines were rolled back to the 3D space (**Fig 3**). Second, after finalizing the 2D stent pattern, we rolled back the vertices of all line segments to the 3D space. Based on the straightforward principle that two points can define a line, this step facilitated a faster rolling back, particularly for lengthy stents. 2D approaches provide operators with a more visually oriented solution for reconstruction work, whilst 3D approaches offer better solutions for full automation, at the cost of visual inspection, manipulation, and validation by an operator, unless ad hoc 3D manipulation tools are available [22–24]. Third, we applied ECG-gating to obtain the IVUS frames at the end diastolic phase for 3D stent reconstruction to lower the lateral and longitudinal motions of the IVUS catheter in clinical cases (swinging effects), which is not encountered in OCT-based 3D stent reconstruction [21–24]. The segmentation from these end diastolic IVUS frames helped in reducing the influence of the lateral and longitudinal motions and fixing the final stent frame in 2D reconstruction (**Fig 4**).

Our proposed methodology presents numerous scientific and clinical applications. In a cardiac catheterization laboratory, it can provide interventionalists with a detailed understanding of the spatial configuration of the deployed stent, pointing out areas of stent under-expansion, strut malapposition, and floating struts at the carina and the side branch ostium. This information is crucial for optimizing stent placement, which is directly linked to improved clinical outcomes [25,26]. It is important to note that for proximal LM, especially with aorto-ostial lesions, where OCT may not work well due to inadequate blood clearance [27], IVUS-based stent reconstruction remains the reliable method, as demonstrated in our clinical case. Additionally, our methodology can be integrated with extended reality techniques to facilitate high-resolution visualization of the deployed stents for educational purposes. It can also enable precise CFD analysis to evaluate the post-stenting local biomechanical environment, focusing on strut level and anatomically sensitive bifurcation areas. Previous studies have associated areas with disturbed flow and high shear stress rates with an increased likelihood of stent restenosis and thrombosis [3]. Identifying these hemodynamically unfavorable areas within the stented regions can further enhance stent placement optimization and clinical outcomes. Moreover, geometrically accurate stent reconstructions can provide valuable feedback to stent manufacturers regarding the design and performance of their products, thus creating opportunities for optimizing stent design. The relatively straightforward steps of our methodology may enable operators without a technical or engineering background (for instance, medical students or fellows) to use it, thereby expanding its applicability.

## 5. Limitations

Despite the potential and promising results, our study does present a few limitations. First, the time needed for manual segmentation on IVUS images could be reduced. For instance, in our clinical case of an 8 mm stent, we spent 120 minutes on the segmentation; this time frame would inevitably increase for longer stents. To address this, we are currently developing a fully automated IVUS segmentation algorithm through neural networks to streamline the process for lumen, struts, and stent contour. Second, the efficacy of our methodology is contingent on the quality of IVUS images. IVUS images with significant image artifacts may hinder the operator’s ability to identify the stent struts. For example, unlike the Eagle Eye Platinum IVUS catheters, which can eliminate the guidewire artifact by housing the guidewire central to its transducer elements, the catheter we used (Opticross 6 HD) induced guidewire-related artifacts. Since the reconstruction technique relies so heavily on the accurate matching and stitching together of IVUS signals, a loss of IVUS signal by guidewire artifacts could affect the final reconstruction, especially if that region happened to be at the unrolling boundaries. Although the Opticross catheter produced guidewire artifacts, this was offset by a higher spatial resolution than the Eagle Eye catheter, which enhanced strut imaging and facilitated a more accurate 3D reconstruction of struts. Third, two stent strategies (e.g., Culotte, T and small protrusion (TAP), etc. [28]) are frequently used for numerous bifurcation lesions, and multiple struts (of the same stent or of two different stents) often overlap in an unpredictable manner. This makes the utility of stent pattern matching less reliable. Although our research methodology has been successfully tested on single stents, a case of two overlapping stents on IVUS represents a technical challenge for our methodology to distinguish between the strut signals from overlapping struts. We aim to address this limitation in our future research. Finally, our 3D reconstructed stents largely featured similar designs, and the stent in the clinical case did not cross the carina since the lesion was an aorto-ostial lesion. Additional studies are planned to test our methodology with various stent designs and bifurcation lesions to further evaluate the versatility and applicability of our approach.

## 6. Conclusion

In conclusion, we have introduced a novel approach that enables precise, replicable, and clinically viable 3D reconstruction of coronary artery stents based on IVUS imaging. Combined with CFD studies, this method has the potential to enhance stent optimization, foster training in stent deployment techniques, and propel research and development within the field of stent design.

## Supporting information

S1 Checklist (DOCX)

S1 Fig Mean stent diameter comparison between 3D reconstruction and μCT reconstruction. (TIF)

S2 Fig Reproducibility analysis for the clinical case, including morphological (a) and mean stent diameter measurement comparison (b and c). The blue tube inside stent model does not depict reconstructed lumen but facilitates better visualization of stent struts.
(TIF)

S3 Fig The distribution of wall shear stress gradient (WSSG) in macroenvironment. In both views (**a**) and (**b**), we can see in the blood flow direction (from proximal to distal), the WSSG (Pa/mm) over the stent struts changed from negative to positive. We can also see the WSSG changes at the carina.
(TIF)
