# Supplementary figures and images for: Experimental validation and clinical feasibility of 3D reconstruction of coronary artery bifurcation stents using intravascular ultrasound
Source: PLoS One. 2024 Apr 16;19(4):e0300098. doi: 10.1371/journal.pone.0300098 (PMC11020600; doi:10.1371/journal.pone.0300098)

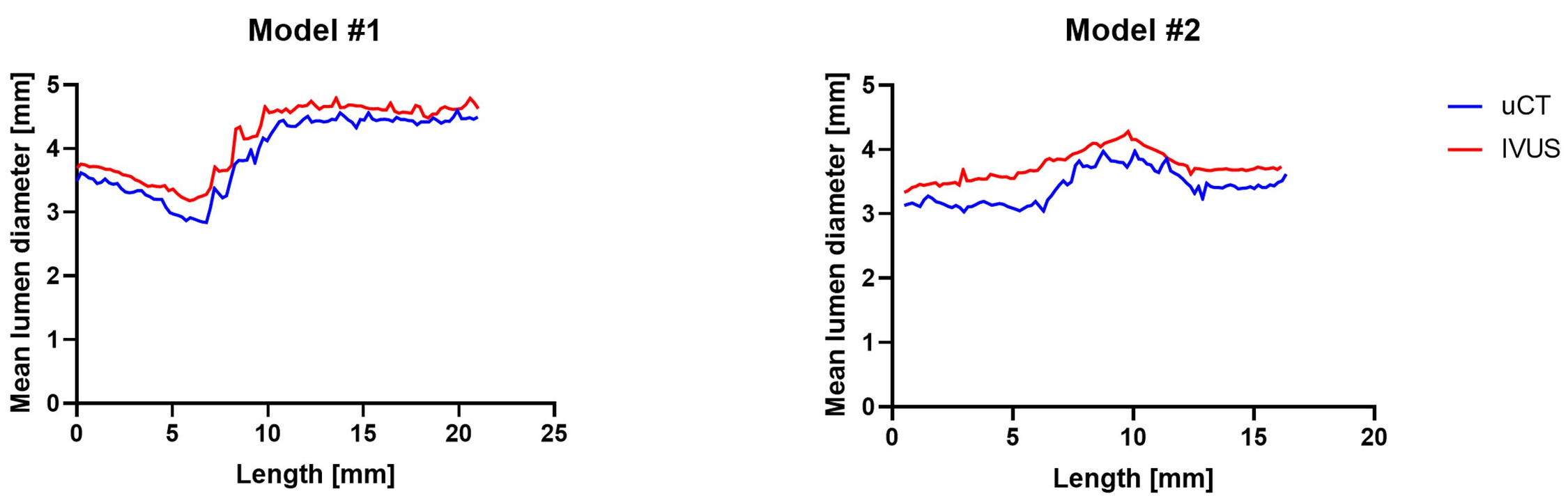

Supplement: S1 Fig — (TIF) [file pone.0300098.s002.tif]

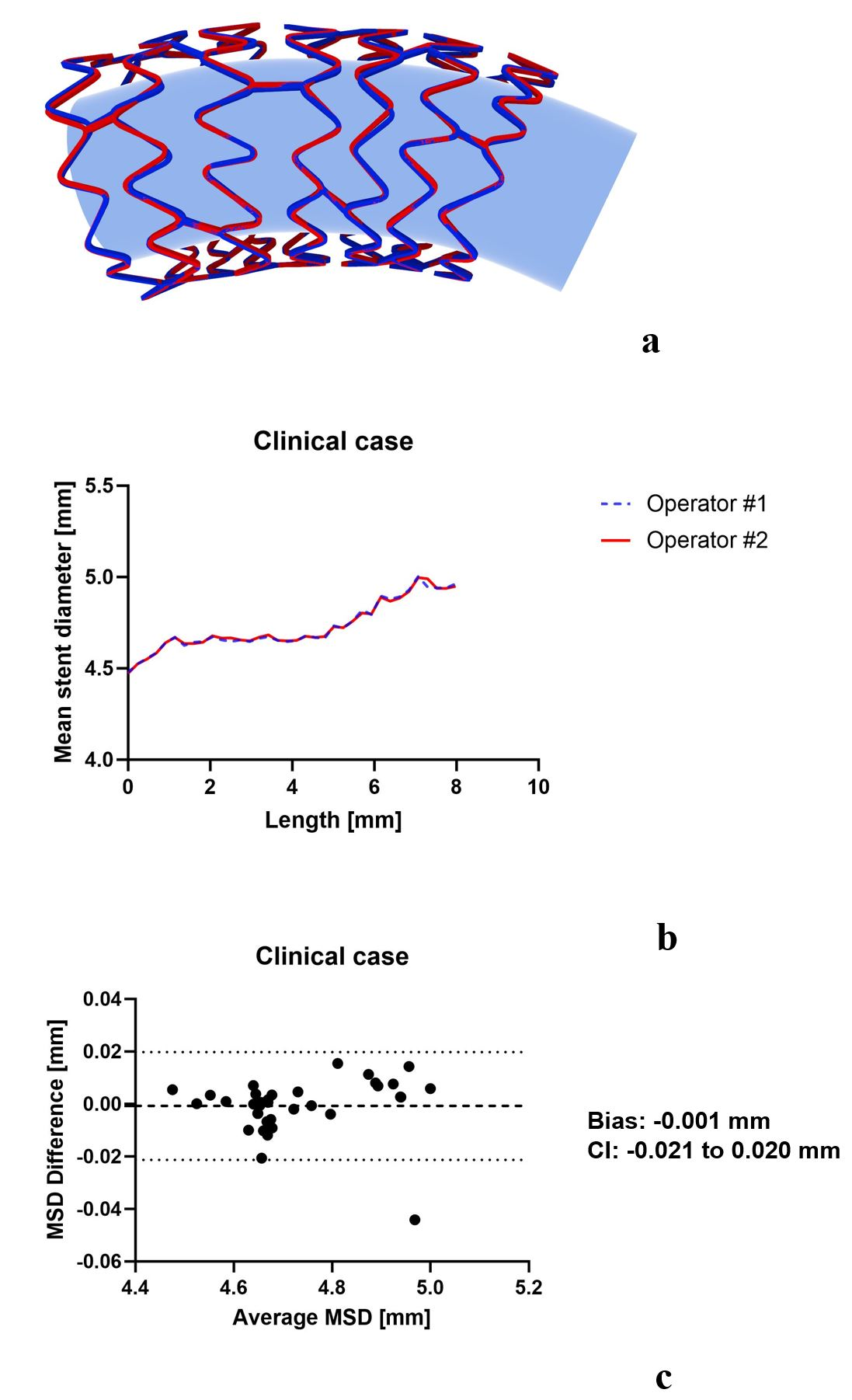

Supplement: S2 Fig — Reproducibility analysis for the clinical case, including morphological (a) and mean stent diameter measurement comparison (b and c). The blue tube inside stent model does not depict reconstructed lumen but facilitates better visualization of stent struts. (TIF) [file pone.0300098.s003.tif]

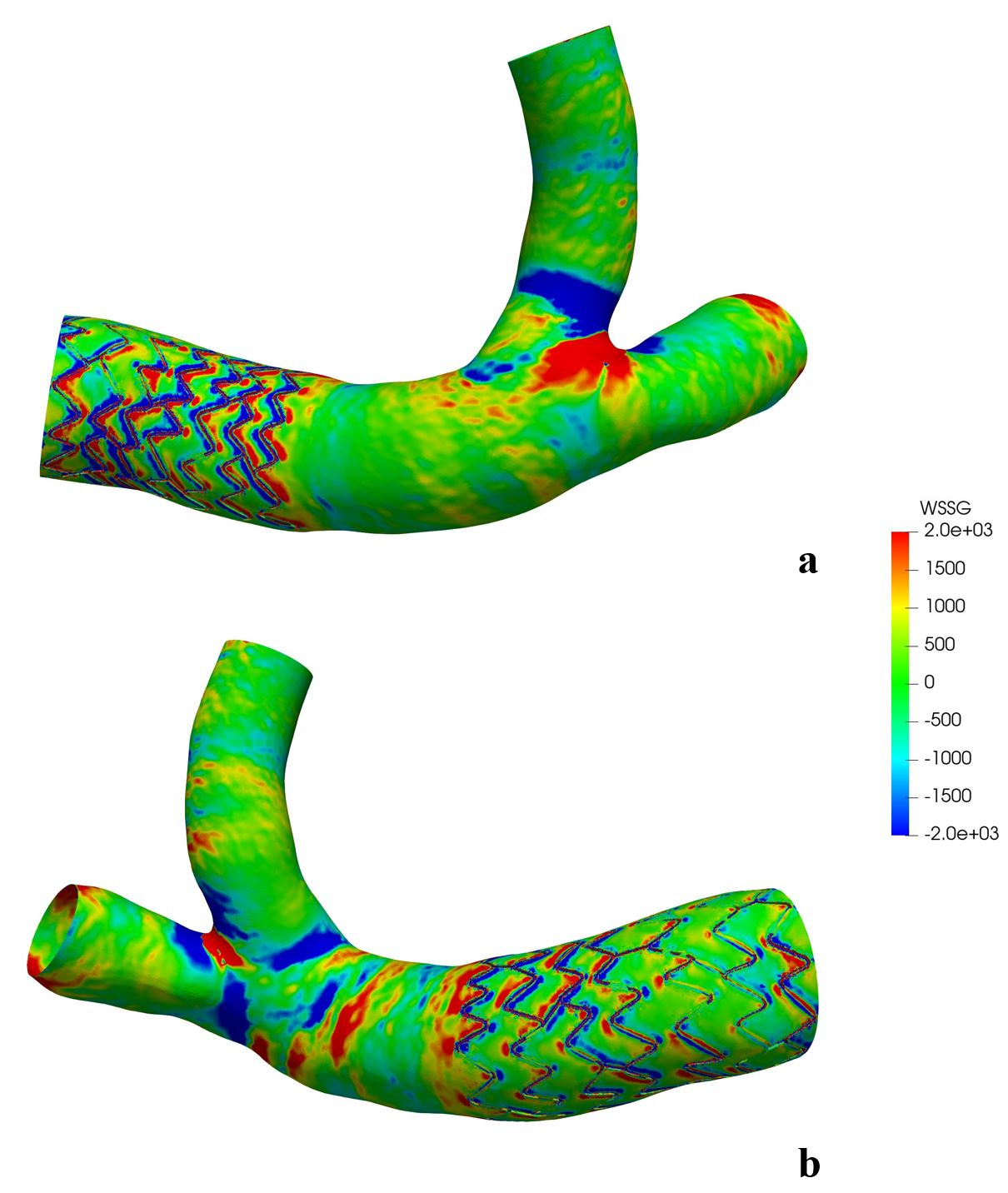

Supplement: S3 Fig — In both views (a) and (b), we can see in the blood flow direction (from proximal to distal), the WSSG (Pa/mm) over the stent struts changed from negative to positive. We can also see the WSSG changes at the carina. (TIF) [file pone.0300098.s004.tif]
